# Studies of the Room-Temperature Multiferroic Pb(Fe_0.5_Ta_0.5_)_0.4_(Zr_0.53_Ti_0.47_)_0.6_O_3_: Resonant Ultrasound Spectroscopy, Dielectric, and Magnetic Phenomena

**DOI:** 10.1002/adfm.201303492

**Published:** 2014-02-01

**Authors:** J Schiemer, M A Carpenter, D M Evans, J M Gregg, A Schilling, M Arredondo, M Alexe, D Sanchez, N Ortega, R S Katiyar, M Echizen, E Colliver, S Dutton, J F Scott

**Affiliations:** Department of Earth Sciences, University of CambridgeCambridge, CB2 0EQ, UK; School of Mathematics & Physics, Queen's University of BelfastBelfast, BT7 1NN, UK; Max Planck Institute of Microstructure PhysicsWeinberg 2, 06120, Halle (Saale), Germany; Institute for Functional Nanomaterials, University of Puerto RicoPO Box 23334, San Juan, 00931–3334, Puerto Rico; Cavendish Laboratory, University of CambridgeMadingley Road, Cambridge, CB3 0HE, UK

## Abstract

Recently, lead iron tantalate/lead zirconium titanate (PZTFT) was demonstrated to possess large, but unreliable, magnetoelectric coupling at room temperature. Such large coupling would be desirable for device applications but reproducibility would also be critical. To better understand the coupling, the properties of all 3 ferroic order parameters, elastic, electric, and magnetic, believed to be present in the material across a range of temperatures, are investigated. In high temperature elastic data, an anomaly is observed at the orthorhombic mm2 to tetragonal 4mm transition, *T*_ot_ = 475 K, and a softening trend is observed as the temperature is increased toward 1300 K, where the material is known to become cubic. Thermal degradation makes it impossible to measure elastic behavior up to this temperature, however. In the low temperature region, there are elastic anomalies near ≈40 K and in the range 160–245 K. The former is interpreted as being due to a magnetic ordering transition and the latter is interpreted as a hysteretic regime of mixed rhombohedral and orthorhombic structures. Electrical and magnetic data collected below room temperature show anomalies at remarkably similar temperature ranges to the elastic data. These observations are used to suggest that the three order parameters in PZTFT are strongly coupled.

## 1. Introduction

There has been recent emphasis upon inventing new room-temperature magneto-electric materials consisting of single-phase multiferroic (containing two or more ferroic order parameters, e.g., ferroelectric and ferromagnetic) materials.^1^,^2^ Until very recently the only such material to exhibit this at room temperature was bismuth ferrite.^3^–^5^ Although attractive for some applications, its electrical conductivity is too high for an ideal component,^5^ and it appears to exhibit only a very weak magneto-electric coupling.^3^,^6^ Strong magneto-electric coupling is desirable for many device applications,^7^–^9^ where the interplay of the magnetic and electrical order leads to the desired behavior, for example in electrically written, magnetically read non-volatile memory elements^10^ or other spintronics and memory applications.^11^,^12^ The search for a high performing single-phase room temperature multiferroic has recently been aided by the report of an additional material in this class.^13^ This material consists of lead zirconate titanate (PZT) with 30–40% Fe and Ta substituted for Zr/Ti at the perovskite B-site^14^–^16^ (PZTFT). This single-phase material exhibits large polarization switching at room temperature produced by modest magnetic fields *H* = 0.3–1.8 T.^13^ In addition, very recently it was shown that electric and magnetic fields can both be used for polarization switching at room temperature.^16^ Other room-temperature multiferroics are also being studied at present, including a Mn-containing Aurivilius-phase oxide.^17^

In the present study, we examine the correlated electro-magneto-mechanical behaviors of Pb(Fe_0.5_Ta_0.5_)_0.4_(Zr_0.53_Ti_0.47_)_0.6_O_3_, using SQUID measurements of magnetization, combined with measurements of electric properties; and changes in elastic properties—from resonant ultrasound spectroscopy—to show that anomalies in one order parameter are strongly associated with anomalies in the other two. We pay particular attention to the regions which show anomalies: ≈40 K, below which the magnetization increases rapidly; a broad peak at 240 K, where a rhombohedral-orthorhombic transformation occurs; and at the orthorhombic-tetragonal transition near 475 K (depending upon Fe concentration). These latter two transitions are already known from the earlier XRD study of Sanchez et al.^14^ The fact that the phase transition sequence elucidated by Sanchez et al. in PFTZT is rhombohedral – orthorhombic mm2 – tetragonal 4mm – cubic Pm3m – exactly as in nonmagnetic BaTiO_3_ – strongly suggests that these transitions are all driven by structural instabilities and not magnetism. The as yet unreported transition at 40 K, reported here, appears to have a much stronger magnetic component. A deeper understanding this transition may well be essential in interpreting the magnetism and electromechanical behavior of PZTFT.

## 2. Results and Discussion

### 2.1. Sample Preparation

Pb(Fe_0.5_Ta_0.5_)_0.4_(Zr_0.53_Ti_0.47_)_0.6_O_3_ ceramic samples were synthesized by conventional solid state reaction route in two steps. In the first step analytically-pure oxides, PbO, ZrO_2_, TiO_2_, Fe_2_O_3_, Ta_2_O_5_, and Nb_2_O_5_ (Alfa Aesar) with purity of 99–99.9%, were used as starting materials. The powders of the respective metal oxides were homogeneously mixed using a planetary high energy ball mill with tungsten carbide media under isopropanol at a speed of 600 rpm for a period of 16 h, then from the resulting slurry the solvent was slowly evaporated until a mixed powder was obtained. For phase formation the resultant powders were calcined at 850 °C for 10 h in a closed alumina crucible. 10% excess of PbO was to compensate Pb deficiency during the high temperature processing. In the second step, the calcined powder was mixed with a 1% poly vinyl alcohol (PVA) solution as a binder and the dried powders were granulated by passing them through a 150 μm-mesh sieve. They were then pressed using a hydrostatic press (3.46 × 10^8^ Pa) into 2 mm thick disk with a diameter of 10 mm. The pressed pellets were heat treated at 600 °C for 2 h for the removal of organic binder, followed by sintering at 1250 °C for 4 h. All heat treatments were performed in air. In order to prevent the PbO loss during high temperature sintering and to maintain the desired stoichiometry, an equilibrium PbO vapor pressure was established by placing PbZrO_3_ along with the samples in a covered alumina crucible. The final samples employed were, in fact, the same ones as in Sanchez et al.^14^ to assure that all data were from the same specimens. In what follows, we have abbreviated Pb(Fe_0.5_Ta_0.5_)_0.4_(Zr_0.53_Ti_0.47_)_0.6_O_3_ as PZTFT4.

### 2.2. X-Ray Diffraction, Electron Microscopy, and X-Ray Spectroscopy

**Figure 1:**
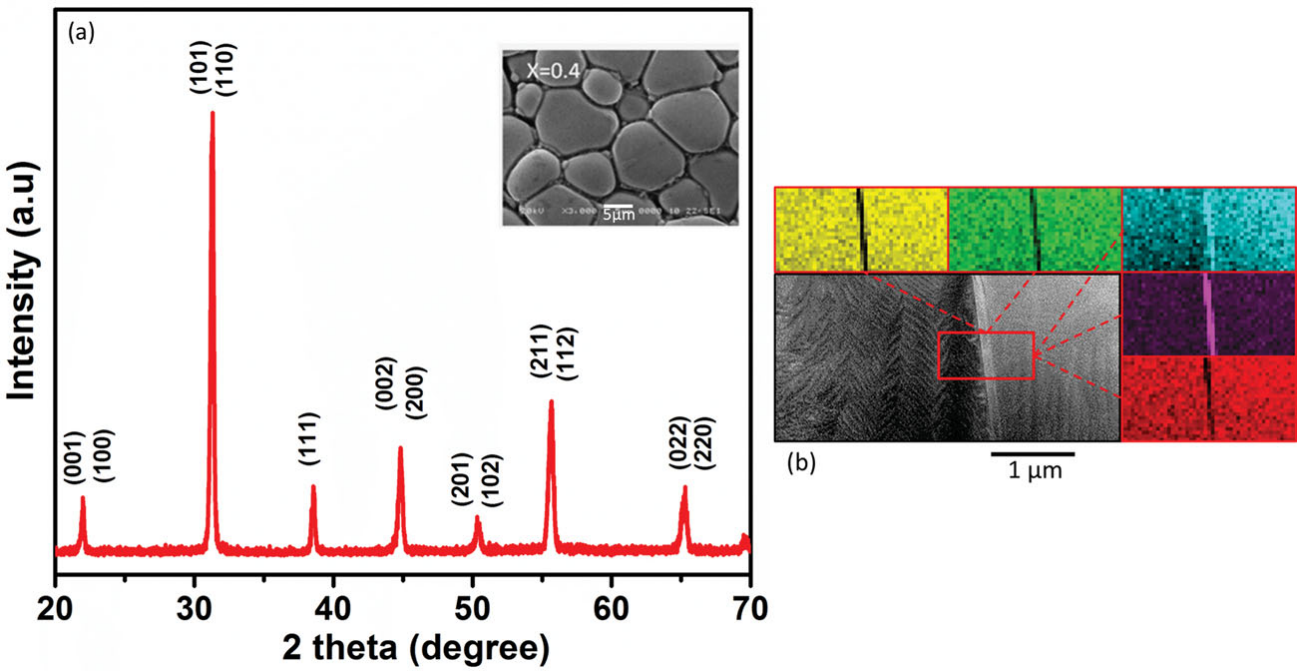
a) Room temperature X-ray spectra of a PZTFT4 ceramic. Inset shows the surface SEM micrograph of the same ceramic. Cross-sectional TEM image of a grain boundary, using a HAADF detector and showing EDX elemental mapping taken across the grain boundary. Yellow, Zr – K; Green, Ta-L; Blue. Pb-M; Purple, Fe-K; Red Ti-K. The bright contrast corresponds to high density regions, suggesting compositional inhomogeneity related to the boundary shown; EDX chemical mapping confirms that Pb, Fe and O elements are present at the grain boundaries.

The phases and purity of the powders and sintered pellets were determined by X-ray diffraction (XRD) (Siemens D5000) using CuK_α_ radiation with wavelength of *λ* = 1.5405 Å. The surface morphology of the (as sintered) pellets of PZTFT4 was studied using a scanning electron microscope (SEM) (JEOL-JSM-5800 at 20 kV) and is shown in **Figure** **1**a.

Figure 1a shows X-ray diffraction patterns for a ceramic sample of PZTFT4. These results suggest a perovskite structure, without the appearance of any secondary diffracting phase (e.g., pyrochlore) within the resolution limit of our equipment (< ≈5%). The diffraction patterns were indexed according to previously reported results for tetragonal PZT. This uniform indexing is a significant result considering that the perovskite B site could be occupied randomly by Ta^5+^, Fe^3+^, Zr^+4^, or Ti^+4^, allowing significant possibility for segregation, even when perovskite phases are formed.

The microstructure of a PZTFT4 ceramic, viewed by SEM, is shown in the inset of Figure 1a. The samples are homogeneous, with grains uniformly distributed throughout the sample surface, the average grain size was found to be nearly 15 μm, with few pores existing in the sintered ceramics. A close examination shows a rather wide boundary region between the grains. It also indicates the segregation of some particles near the grain boundaries which is likely to be linked to a material of a different composition to the main phase, decorating the individual grains.

The morphology and microstructure of the grains and the grain boundaries were further investigated using transmission electron microscopy (TEM) and energy dispersive X-ray analysis (EDX). The nature of the material at the boundaries was determined by the following method. First, lamellae were cut which were centered on a grain boundary, using a Focused Ion Beam (FIB, FEI200TEM FIB) following a method outlined by Schilling et al.^18^ Once the FIB had been used to cut a lamella free, a sharpened glass needle was used to lift the lamella clear of the bulk ceramic and place it on a holey carbon (TEM) grid. Once on the grid, the chemical composition of the area between the grains was investigated using a field emission TEM (FEGTEM using a FEI Tecnai F20 field emission gun operated at 200 kV, with EDX capability). High-angle annular dark-field (HAADF) images and concomitant spectroscopy data were collected, as shown in Figure 1b.

The chemical analysis indicated that while the grain composition remains constant (PbZr_0.296±0.004_ Ti_0.168±0.004_ Fe_0.174±0.002_ Ta_0.272±0.008_ has already been reported),^13^ there is a clear inhomogeneity at the grain boundaries, especially for the elements Ti, Zr, Fe, and Ta, while no apparent change can be observed for larger Pb cations. For example, in Figure 1b, an increased concentration of iron is clearly seen at the grain boundary, accompanied by Ti deficiency. To investigate whether the changes observed in composition at the grain boundaries result from a coherent second phase, or from decoration of grain boundaries with mixed or amorphous phases of no fixed composition, additional samples were prepared across different grain boundaries. These were further investigated with a Cs-corrected STEM at 300 kV. The results of this investigation suggested that there is no presence of a specific Fe-rich secondary phase. Only some of the observed grain boundaries showed the compositional inhomogeneity shown in Figure 1b, while others were relatively free from segregation.

Therefore, while a contribution from the secondary material in the grain decorations to the magnetic signal can not be quantitatively ruled out, it is known from XRD that any possible impurity phase cannot be <≈5%. No coherent second phase was detected in the small number of grain boundaries examined with EDX or TEM. Highly magnetic secondary phases such as Fe_2_O_3_ and Fe_3_O_4_ can be ruled out as significant contributors, as no signature of their characteristic transition temperatures is observed (see below). Furthermore, the intimate connection between the magnetic and electrical order parameters in PZTFT in FIB cut single crystals, with no grain boundary region whatsoever, has been demonstrated in Evans et al.^13^ as well as discussed in as-yet unpublished work presented at a recent conference^19^ and these results are in agreement with the information presented here.

### 2.3. Magnetization

Magnetic measurements were carried out using a Quantum Design Magnetic Properties measuring system (MPMS). The magnetic susceptibility, *χ* =*M*/*H*, as a function of *T* was measured during heating, after cooling both in zero-field (ZFC) and in the measuring field (FC) of 0.1 and 5 T. Isothermal magnetization measurements were also made, –5 ≤ *H* ≤ 5 T, at 5, 150, and 300 K. In all cases, measurements were made on small pieces of sintered pellets

**Figure 2:**
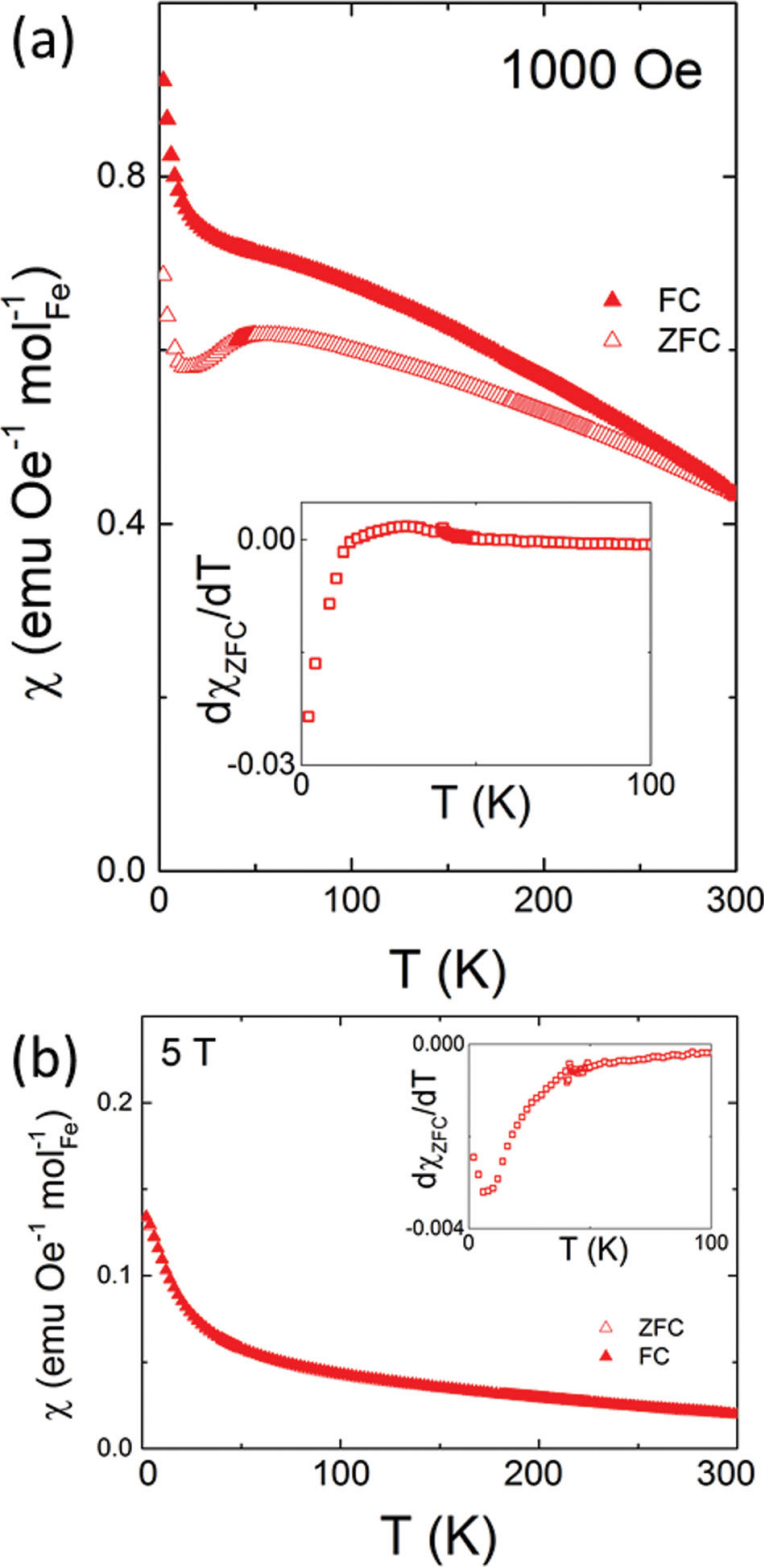
a) Magnetic susceptibility of PZTFT4 collected during heating, after cooling in zero field, *χ*_ZFC_, (open symbols) and in the measuring field of 1 kOe, *χ*_FC_ (closed symbols). d*χ*_ZFC_/dT is inset for both compositions. b) Magnetic susceptibility PZTFT4 after cooling in zero field, *χ*_ZFC_, (open symbols) and in the measuring field, *χ*_FC_ (closed symbols) of 5 T.

The magnetic susceptibility, *χ*, of PZTFT4 is shown in **Figure** **2**a. An irreversibility between *χ*_ZFC_ and *χ*_FC_ over the entire *T* range is observed; at the maximum *Tχ*_ZFC_ and *χ*_FC_ appear to be converging, indicating that the FM correlations are suppressed at a hypothetical Curie temperature (*T*_C_) just above 300 K. Although no well-defined definition of the Curie temperature exists for materials exhibiting cluster magnetism, the convergence of the FC and ZFC curves signifies the upper limit temperature of magnetic poling. Magnetic measurements to higher *T* would further confirm this estimate of *T*_C_. At intermediate *T* a transition is observed in PZTFT4. This broad peak has a maximum at *T*_f_ ≈ 50 K, and below this *χ*_ZFC_ decreases whereas *χ*_FC_ continues to increase; this may indicate spin or cluster glass ordering. On further cooling a more dramatic increase in *χ* is observed below 10 K. Consideration of d*χ*_ZFC_/d*T*, inset Figure 2a, shows that the change in the *T* dependence of *χ*_ZFC_ occurs at ≈24 K. This feature may arise from uncompensated spins which align with the field on cooling to sufficiently low *T*. At higher fields, Figure 2b, the glassy transition observed at low field is suppressed, as is the increase in *χ* from uncompensated spins at low *T*. At high fields *χ*_ZFC_ and *χ*_FC_ are reversible.

Note that if any significant volume of the highly magnetic Fe_2_O_3_ or Fe_3_O_4_ phases were present, their characteristic transitions would be observed. These are the Morin transition in Fe_2_O_3_ at 260 K and the Verwey transition in Fe_3_O_4_ at 120 K. Neither of these are observed.

**Figure 3:**
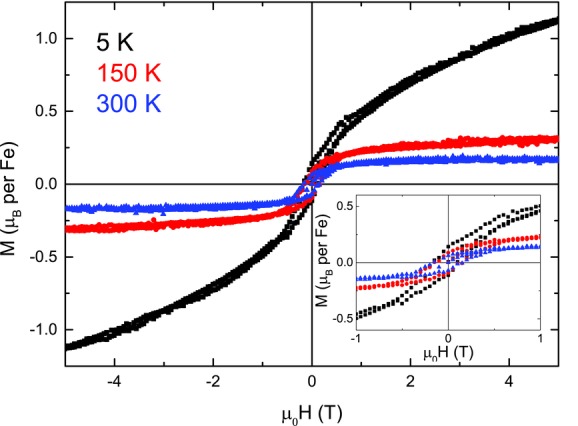
Isothermal magnetization of PZTFT4 at 5 K (black), 150 K (red), and 300 K (blue). The low field region is shown in greater detail as an inset.

The isothermal magnetization measurements, *M*(*H*), **Figure** **3**, are consistent with the magnetic ground states described above. At *T* ≥ 150 K, the magnetization saturates to a low value on application of a small magnetic field. The shape of *M*(*H*) is characteristic of ferromagnetic or ferrimagnetic ordering, although the small saturation value, 0.2–0.3 μ_B_/Fe (0.65–0.97 emu/g), is significantly less than would be anticipated for a fully polarized Fe^3+^ system (*M*_SAT_ = *gS* = 5 *μ*_B_/Fe). At 5 K and low field (*μ*_0_*H* < 1 T) the shape of the *M*(*H*) changes dramatically and irreversibility is still observed. In higher fields, however, the magnetization does not saturate and is still increasing at the limiting field of 5 T. The shape of the low *T* isothermal magnetization is consistent with the formation of a spin or cluster glass on cooling *T* < *T*_f_.

### 2.4. Dielectric Examination

**Figure 4:**
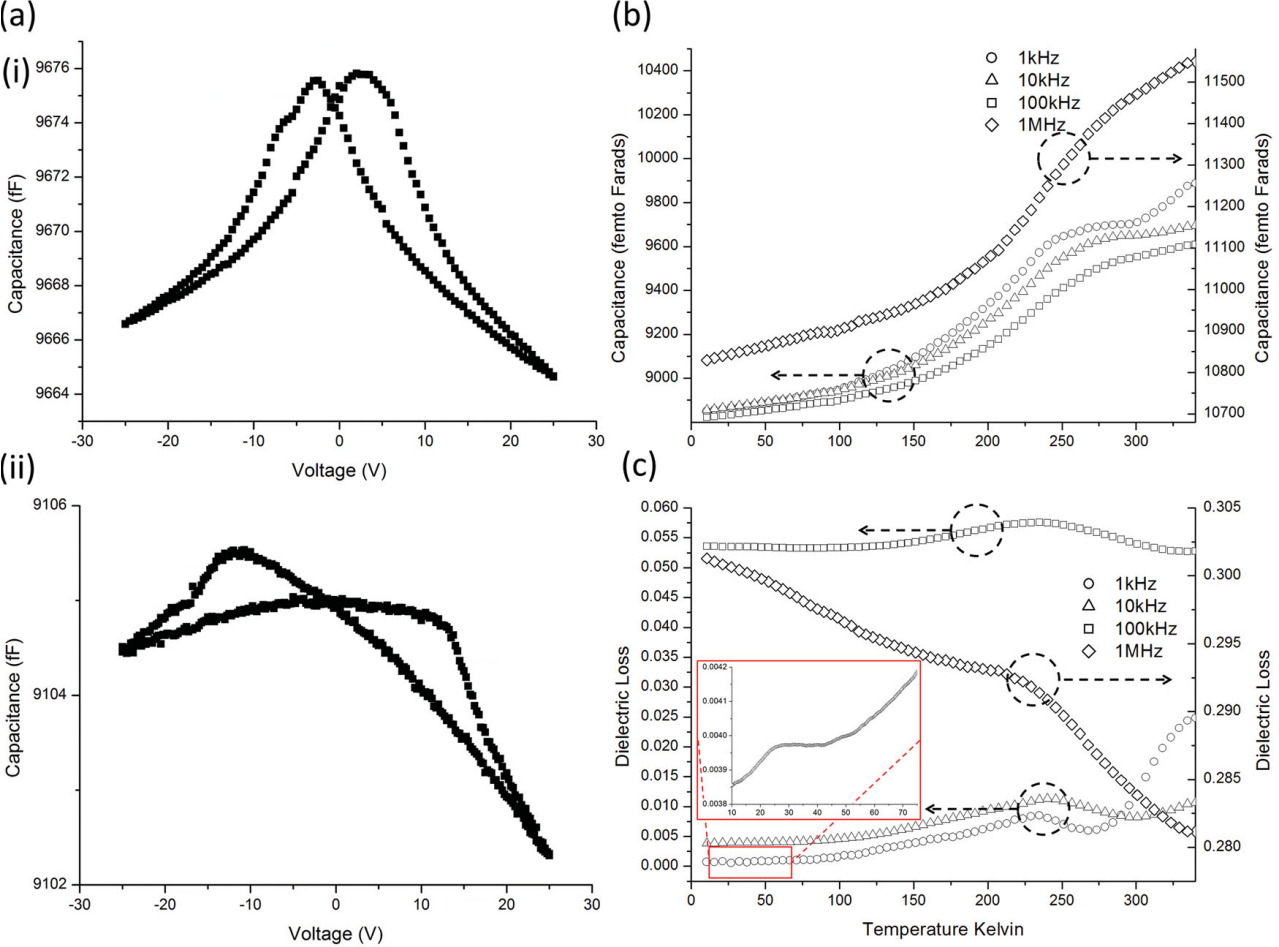
Dielectric data taken during cooling. a) Capacitance-voltage plots are shown at i) 300 K and ii) 150 K. Two peaks in the capacitance are evident at 300 K and 150 K showing the samples are ferroelectric. b,c) dielectric data, showing electrical behavior as a function of temperature for frequencies of 1 kHz, 10 kHz, 100 kHz, 1MHz with b) capacitance and c) loss tangent. Broad peaks are visible in both capacitance and loss all frequencies at ≈240 K. In (c), the lower frequency (1 kHz and 10 kHz) dielectric loss tangent generally decreases with decreasing temperature with clear peaks at ≈240 K and more subtle anomalies at ∼35 K (inset). The higher frequency data (100 kHz and 1 MHz) do not decrease with decreasing temperature, with the 1 MHz data actually increasing, although both still display a peak at 240 K and slight peaks at 35 K. Because dielectric loss in the absence of phase transitions generally decreases with decreasing temperature, it is likely that the 100 kHz and 1 MHz data are influenced by the experimental set up in some way.

In order to get clean results on fully dense sample material, a dielectric investigation was performed on lamellae cut from a single grain, within a co-planar capacitor geometry. While this allows the collection of dielectric data without contributions from outside of the primary phase, the small signal generated also results in a small background signal from the experimental setup itself. This background is expected to be effectively independent of temperature. To ensure that the results were meaningful, CV loops were collected, **Figure** **4**a, before and after the temperature sweeps, with no discernible change. This confirms: i) the sample is indeed ferroelectric, although there is clearly a large background contribution; ii) the temperature dependent measurements did not change the properties of the sample.

In order to get clean results on fully dense sample material, a dielectric investigation was performed on lamellae cut from a single grain, within a co-planar capacitor geometry. While this allows the collection of dielectric data without contributions from outside of the primary phase, the small signal generated also results in a small background signal from the experimental setup itself. This background is expected to be effectively independent of temperature. To ensure that the results were meaningful, CV loops were collected, Figure 4a, before and after the temperature sweeps, with no discernible change. This confirms: i) the sample is indeed ferroelectric, although there is clearly a large background contribution; ii) the temperature dependent measurements did not change the properties of the sample.

The method used for lamellae preparation is based on the work done by Schilling et al.^18^ where a Focused Ion Beam (FIB, FEI Nova 600) is used to cut a lamella from an individual grain of the bulk ceramic. These lamellae are then lifted from the bulk, using a sharpened glass needle, and placed on a platinized magnesium oxide substrate, which had previously been patterned into a capacitor structure. Once in place the sample was annealed at 600 °C for 1 h which causes gallium, introduced by the FIB milling, to rise to the surface. Once the expunged gallium/gallium oxide is at the surface, it is removed by acid etching (5 mins in 2.8 mol L^-1^ HCl) in a method pioneered by McGilly et al.^20^ It's worth noting that it was observed that annealing at temperatures of 650 °C, or greater, caused the samples to decompose. After the acid etch, platinum was deposited on the areas of the lamellae over the electrodes, also using the FEI Nova 600. This is the procedure used by McQuaid et al.^21^ in their co-planar barium titanate capacitor structures to improve electrical contact between the lamellae and the electrode pads.

Individual lamellae plane normals were in the [110] direction and all measurements were made in this plane. The capacitance-voltage loops at 300 K, Figure 4a, are typical of a lamella in a co-planar geometry. The slight asymmetry in 300 K CV loop, that is, capacitance at +25 V having lower value than at –25 V, is likely to be an experimental artifact, because the lamella is only placed on the sputtered platinum and hence any topography in ether surface will prevent a perfect contact. A doubly peaked ferroelectric CV loop is still obvious in the 150 K measurement. Note that the two peaks have moved apart to ±15 V rather than the ±5 V at 300 K, which is expected due to the normal increase of coercive field with temperature difference from the Curie temperature (i.e. decreasing temperature below T_C_). We also observe that the height of the CV loop has diminished from ≈9 fF at 300 K to ≈1.5 fF at 150 K. This reduction in capacitance with decreasing temperature is similar to the lowering of relative permittivity (away from transition temperatures) observed in BaTiO_3_,^22^ which follows an analogous sequence of phase transitions. 10 K data showed no obvious peaks in the capacitance and only hints of hysteresis near the limit of experimental resolution; this is likely to be due to a combination of the coercive field increasing and the relative permittivity decreasing with decreasing temperature.

After the capacitance voltage loops were taken, dielectric data were collected during cooling between 340 and 10 K at frequencies of 1 kHz, 10 kHz, 100 kHz, and 1 MHz, as shown in Figure 4b,c. Both the real *ε*′(*T*) and imaginary (loss *ε*″(*T*)) components of the permittivity reveal clear changes in slope at 240 K, the temperature previously assigned as the rhombohedral-orthorhombic phase transition.^14^ There also appears to be a subtle anomaly at ≈35 K, which is manifested in the loss (best viewed in the 10 kHz and 100 kHz data) by a broad peak. This 35 K anomaly isn't visible in the capacitance data, possibly due to the drastically reduced signal at low temperatures.

The permittivity values measured were not coincident during heating and cooling cycles. This was originally assigned to drift, a distinct possibility when measuring fF. However, in light of the magnetic data of Section 5, and the more robust elastic data of Section 2.5.2.1, it seems possible that there is thermal hysteresis in the dielectric measurements.

### 2.5. Resonant Ultrasound Spectroscopy (RUS)

#### 2.5.1. Experimental Methods

The sample of PZTFT4 used for ultrasonic measurements was a small, asymmetric sample, weighing 8.6 mg. This sample was used initially but broke in two, and a 5.0 mg piece was used subsequently. RUS spectra were collected in situ at low and high temperatures. The low-temperature instrument utilizes an Orange He-flow cryostat, with DRS M^3^ODULUS II electronics, as described by McKnight et al.,^23^ and the sample chamber is filled with a few mbar of helium. The high temperature instrument utilizes a horizontal tube furnace, into which are inserted alumina buffer rods, as described separately by McKnight et al.,^24^ and the sample is heated in air. Temperature calibration in the high temperature instrument has been checked against the α ↔ β transition in quartz (846 K), and quoted temperatures for both instruments are believed to be accurate to within ±1 K. Data collection is automatic in cooling and heating sequences, with a settle time of 20 minutes at each temperature to allow for thermal equilibration. Raw spectra are routinely transferred to the software package IGOR PRO (Wavemetrics) for analysis. Selected peaks are analyzed by fitting with an asymmetric Lorentzian peak function, in order to determine the peak frequency, *f*, and peak width at half maximum height, Δ*f*. Elastic constants scale with *f*^2^ and the mechanical quality factor, *Q* = *f*/Δ*f*, is a measure of acoustic dissipation. Resonance modes of a small sample are dominated by shearing motions and, for a polycrystalline sample, provide information about the shear modulus. Use of a rectangular parallelepiped allows determination also of the bulk modulus, from fitting to the frequencies of a number of peaks, but an irregularly shaped sample can still be used to follow the temperature dependence of the shear modulus.

#### 2.5.2. Results

##### 2.5.2.1. Low Temperature

**Figure 5:**
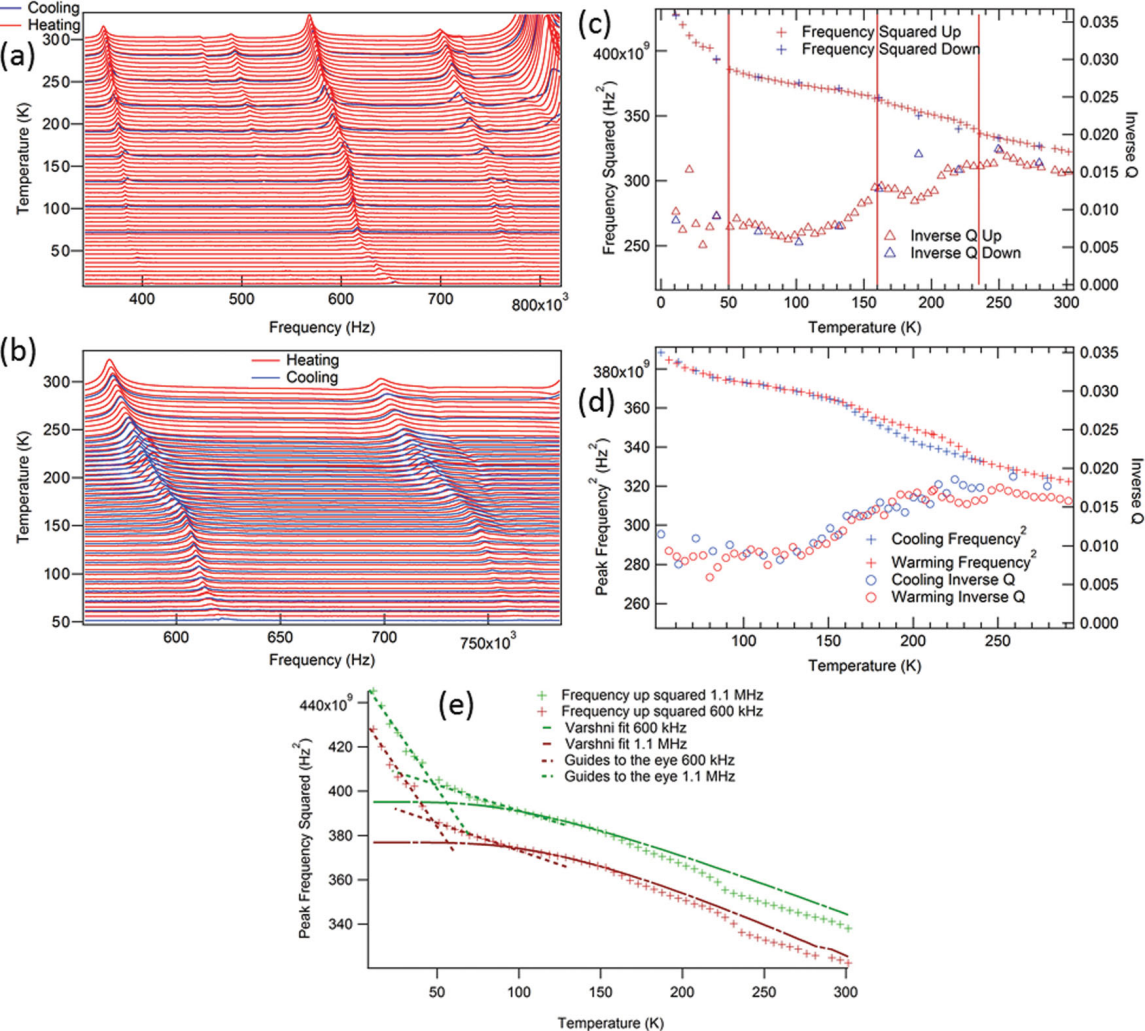
Low temperature RUS data. a) Segments of RUS spectra stacked in proportion to the temperatures at which they were collected. Note the breaks in trend of reducing resonance peak frequency (elastic softening) with increasing temperature at ≈50 K, ≈160, and ≈235 K. b) Segments of RUS spectra collected in a second cooling and heating run to characterize the hysteretic region between ≈160 and ≈235 K. In this 75 K interval resonance peaks occur at lower frequencies during cooling than during heating. c) Results of peak fitting for the peak in figure 5a with frequency near 565 kHz at room temperature. Vertical lines indicate the proposed Néel point and limits of the hysteresis. d) Results of peak fitting from the resonance peak in figure 5b with frequency near 565 kHz at room temperature, showing the hysteresis region in detail. e) Depiction of the deviations of the squared peak frequencies from the expected trend given by Varshni.^25^ Guides to the eye are given to show where deviations occur at ≈90 K and ≈50 K. Data for the 1.1 MHz peak are scaled to be close to that of the 600 kHz peak.

Segments of RUS spectra collected in the frequency range 100–1200 kHz, with 65 000 data points per spectrum during cooling in 30 K steps and heating in 5 K steps between ≈5 K and ≈300 K are shown as a stack in **Figure** **5**a,b. The left axis is actually amplitude in volts from the amplifier but the spectra have been offset in proportion to the temperature at which they were collected and the axis is labeled in Kelvin. With increasing temperature, all the resonance peaks shift to lower frequencies (elastic softening) but with clear breaks in trend. In particular there is a change of slope at ≈50 K, corresponding to the transition observed in dielectric and magnetic mesurements and hypothesised to be due to magnetic ordering/spin glass behavior, and two smaller changes in trend at ≈160 and ≈235 K. There is also evidence of hysteresis between the latter two temperatures as can be seen from the heating and cooling traces, which was confirmed in a second cooling and heating run with smaller temperature intervals (Figure 5b).

Figure 5c,d show the results for *f*^2^ and *Q*^−1^ obtained by fitting to a resonance peak which has a frequency of ≈565 kHz at room temperature. The break in slope at ≈50 K corresponds to an increase of the shear modulus with falling temperature through the magnetic ordering temperature. Another apparent break in slope exists at ≈90 K, away from the trend to decreasing slope in peak frequency variation that is expected upon cooling, as described by Varshni^25^ and further examined in the discussion, below. Slight stiffening below ≈235 and ≈160 K is confirmed (in heating and cooling respectively). Vertical lines have been added to Figure 5c to mark where these breaks in trend occur. The hysteresis is clearly evident in data from the same peak obtained in the second cooling/heating sequence (Figure 5d). Relatively low values of *Q*^−1^ (≈0.005) persist from ≈5 K to ≈150 K, and, if there is any variation through the magnetic ordering temperature, it is below the level of noise. There is then a continuous increase between ≈150 and ≈230 K, reaching relatively high values of ≈0.015 (Figure 5c,d). The high value at 20 K is likely to be an artifact due to the very low signal to noise ratio at that temperature.

##### 2.5.2.2. High Temperature

**Figure 6:**
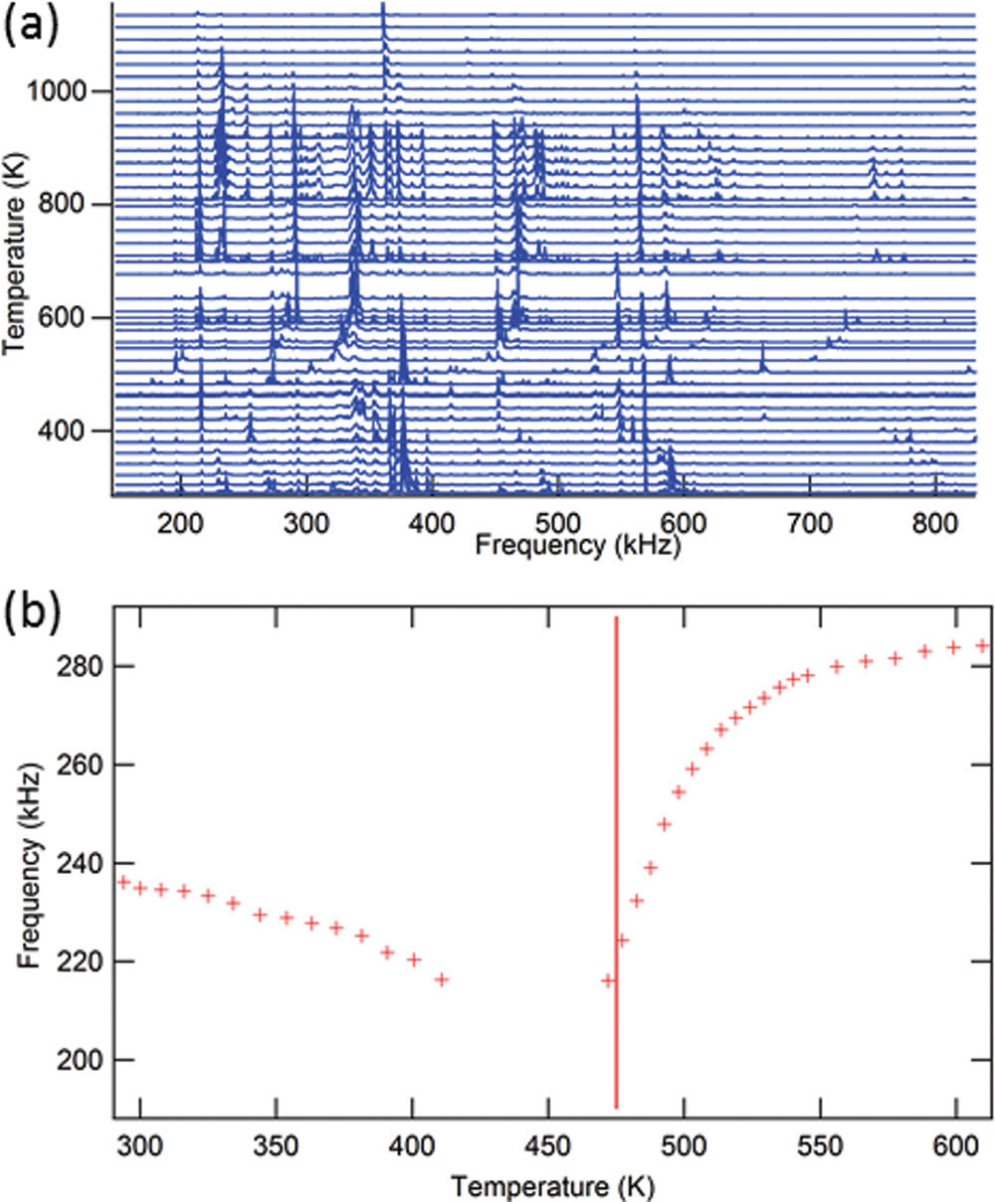
a) Segments of RUS spectra collected during cooling in the high temperature instrument. As with Figure 5a,b, the left axis is amplitude but the spectra are shifted in proportion to the temperature at which they were collected and the axis labeled accordingly. Relatively sharp resonance peaks which do not shift in frequency with changing temperature are from the alumina rods; weaker peaks which move to a clear minimum near 472 K are from the sample. b) Temperature dependence of frequency for a single resonance peak in Figure 6a. Peaks could not be detected in spectra collected at 5 K steps in the interval between 405 and 472 K. The vertical line indicates 475 K, where the O–T transition was determined to occur by Sanchez et al.^14^

Resonance peaks in spectra obtained from the high temperature instrument are always weaker than those from the low temperature instrument because the sample sits between the ends of alumina rods, rather than directly between the transducers. With acoustic losses corresponding to values of *Q*^−1^ as high as ≈0.015 at room temperature, fitting of individual peaks becomes difficult. Nevertheless, the stack of spectra superimposed from both heating and cooling sequences, shown in **Figure** **6**a, clearly shows the trends of both frequency and acoustic loss. Relatively sharp peaks which have nearly fixed frequencies are from the alumina rods while the weaker, temperature-dependent peaks are from the sample. The latter are seen most clearly where they interact with rod peaks. With increasing temperature resonance peaks of the sample shift to lower frequencies, reaching a minimum at ≈472 K. They then recover to higher frequencies, with a plateau of nearly constant values between ≈650 and ≈950 K, before softening again. The sample peaks disappear entirely between ≈405 and 472 K, indicating an increase in acoustic loss in this interval, but they sharpen immediately above this, indicating a significant and abrupt decrease in *Q*^−1^. They then become broader above ≈800 K, when the return to a softening trend becomes re-established. The sample suffered weight loss and a change in color, presumed to be by vaporization of lead, when heated to temperatures above ≈1100 K. The durability of the material heated in air at these temperatures appears to be poor.

Figure 6b shows the temperature dependence of frequency for a single resonance peak from the sample. The pronounced softening as *T* → ≈472 K from above and below is evident. A vertical line has been added to mark the position of the T ↔ O transition temperature found by Sanchez et al.^14^ No data points are shown between 405 and 472 K, because of the complete attenuation of resonances in this interval.

#### 2.5.3. Discussion

The key feature of elastic and anelastic properties is that they are indicative of the role of strain in phase transitions. In particular, dynamic relaxational effects give rise to Debye-like features in the measured elastic constants and acoustic loss and phase transitions with an order parameter coupled to strain generally cause elastic softening of the low symmetry structure. Given that the resonant modes are determined largely by shearing motions, the insights provided by the RUS data presented here are into the influence, primarily, of shear strains. In this context, the lowest temperature anomaly, below ≈90 K, involves slight stiffening with falling temperature. There is no associated peak in *Q*^−1^ which might have indicated some freezing process, say of oxygen vacancy motion or of ferroelastic twin walls. The magnetic data imply some change in magnetic ordering behavior but if this was coupled with any significant shear strain it would be expected to give rise to softening in the usual way via coupling terms of the form *λ*_1_*em*^2^, where e is a shear strain, *m* is the magnetic order parameter and the coupling coefficient, *λ*_1_, defines the strength of coupling. If this coupling is small, however, stiffening due to terms of the form *λ*_2_*e*^2^*m*^2^ can occur and this seems to be the most likely explanation of what is observed. The data can be interpreted in terms of two separate transitions, at ≈50 and ≈90 K or in terms of a single anomaly starting at ≈90 K. The Varshni equation gives a baseline in Figure 5e and dashed lines show two stiffening trends. In light of the magnetic data from the same sample which have been interpreted (above) as showing an antiferromagnetic ordering transition at 50 K, the preferred explanation for the moment is that the elastic anomaly is due to biquadratic coupling between a shear strain and the magnetic order parameter. The trend below 90 K could then be a precursor effect but additional characterization of the structure is required before any definitive conclusions can be drawn about this. Stiffening with this form has been seen in Bi_0.9_Nd_0.1_FeO_3_ where it was certainly associated with antiferromagnetic ordering.^12^

Also on the basis of other measurements, the anomalies in elastic and anelastic properties near 200 K are attributed to the O ↔ R transition in which there is a change in orientation of the ferroelectric displacements. This transition point was been put at 241 K on the basis of dielectric measurements. In the region of hysteresis, between ≈160 and 235 K, the shear modulus stiffens as the transition is traversed from high temperature to lower temperature, and there is no clear softening behavior and no sign of the sharp minimum that has been observed at the O ↔ R transition in BaTiO_3_ (BT).^23^,^26^ Higher loss in the orthorhombic phase than the rhombohedral phase is, however, similar to that seen in ceramic BaTiO_3_ with RUS.^23^ This transition is necessarily first order in character and must involve some temperature interval of coexisting phases which might account for a smoothing of elastic properties if the two phase field actually extends through most of the temperature interval between 160 and 235 K. The data are perhaps more similar in form to what is seen at the analogous transition in K_1-x_Na_x_NbO_3_ (KNN),^27^ though the hysteresis limits are marked by abrupt changes in shear modulus indicative of a very narrow two phase interval. KNN undergoes both ferroelectric and octahedral tilting transitions.

The temperature of the measured high temperature phase transition is in good agreement with the value of 475 K given by Sanchez et al.^14^ for the T ↔ O transition temperature from XRD and Raman measurements. In their report, Sanchez et al.^14^ also use Raman spectroscopy to attempt to locate the temperature at which the material becomes cubic. The Raman data show a disappearance of all lines, compatible with a cubic transition, near 1123 K, but the polarization anomaly data suggest a higher temperature of approximately 1300 K. A dramatic increase in *Q* is expected in the cubic phase, plus stiffening of the shear modulus with increasing temperature, but neither of these is observed in the RUS data collected between 475 and 1150 K. According to the present observations, therefore, the T ↔ C transition point must be above at least 1150 K.

## 3. Summary

As outlined in previous sections, PZTFT exhibits anomalies at comparable temperatures, within ≈15 K, for multiple types of order. As all of the measurements were taken separately, that is, only one properties was measured at a time, an examination of the same temperature range for three different properties, each representing a different order parameter, may be illuminating. The pellets used to collect magnetic susceptibility had previously been heated to ≈900 °C for RUS, which, while far below the synthesis temperature of 1250 °C, may be sufficient to cause some irreversible effects. However, as described in this section, the correlations between the magnetic data and the elastic data (taken before heating) and dielectric data (taken on individual lamellae from pellets in the same batch) are robust.

**Figure 7:**
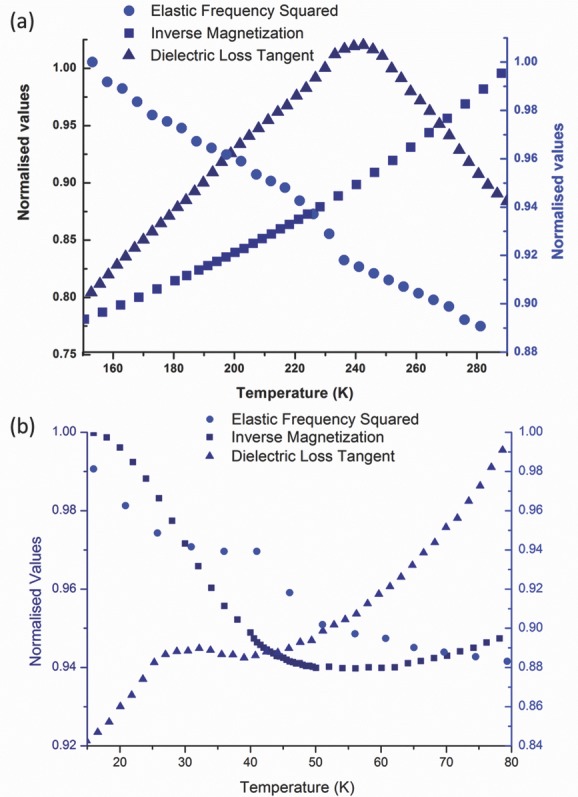
Variations in the three distinct types of order present in PZTFT4 at two transition regions, with data collected during heating. a) Clear changes in both dielectric loss tangent and elastic frequency are observed at ≈240 K, with a peak in the loss tangent and a step-like feature in the squared elastic frequency. b) Magnetic, elastic, and dielectric anomalies all occur in the region of 35-50 K, with a small peak in the dielectric loss tangent, a steep change in slope in the inverse magnetization and a change in slope in the squared elastic frequency (cf. Figure 5e).

In **Figure** **7**a, we see a very pronounced peak in the dielectric loss tangent at ≈240 K. This is likely to be associated with the known orthorhombic–tetragonal phase transition. At the same temperature, the normalized elastic frequency squared shows a pronounced change at 235 K (Figure 7a), a very similar position to the peak in the dielectric loss. The behavior of the magnetic susceptibility here is obviously changing, although no clear anomaly at 235–240 K is present (Figure 7a). This transition shows hysteresis in both elastic and dielectric measurements, indicating a first order character.

The lower transition between ≈30 K and 50 K, shows an even stronger correlation between the temperatures of disturbance of the order parameters. The dielectric loss tangent shows a general smooth decrease with cooling but displays a superimposed peak (Figure 7b). The deviations from the smooth cooling trend start at ≈45 K, the loss then peaks at ≈30 K and returns to the trend at just below 20 K. The peak in the dielectric loss is much smaller than that observed in the 245 K data, but given the freezing in of the capacitance voltage loops (Figure 4a) a smaller dielectric response is not unexpected. The elastic frequency also shows a change in this temperature range (Figure 7a); the most notable change in gradient appears at ≈30 K, where the elastic frequency increases sharply with cooling temperature. However, there is also a more subtle anomaly at ≈45 K were the gradient of the squared frequency changes.

There is a dramatic change in the gradient of the inverse magnetization at ≈45 K, with a minimum in the inverse magnetization at almost exactly the same temperature as the anomalies observed in elastic and dielectric data. Temperature dependent changes in the strength of magnetization are obvious from differences in MH loops taken at 300 K and 5 K (Figure 3), although MH behavior in the intermediate 35–50 K region has not been examined. In these MH measurements, the shape of the loops change slightly with temperature, with a sloping signal superimposed on the ferromagnetic hysteresis at low temperature, while the remnant magnetization is significantly higher at 5 K than at 300 K.

The occurrence of coincident anomalies implies that coupling terms of significant magnitude betwee order parameters exist in the Landau expansion. If this were not the case, anomalies could be observed in one order parameter with no change in the others, unlike what is observed. Due to a lack of knowledge of the space groups above and below the transition, it is not possible to write down the complete Landau expansion at this stage. However, as has been stated above, because there is elastic stiffening associated with the cooling rather than softening we might expect a term of the form *λ*_2_*e*^2^*m*^2^ to dominate over the *λ*_2_*em*^2^. As the dielectric data has anomalies at the same temperature and Evans et al.^13^,^16^ show that a magnetic field can change the ferroelectric domain state, that is, coupling exists, it seems likely that there will be a significant term with an electrical polarization component as well. There is an equivalence between of magnetic and electric fields in this material with respect to switching.^16^ The coupling that leads to switching of polarization P with applied field *H* may involve strain, e, and magnetostriction *m^2^e^2^*, or direct magnetoelectric terms such as *α_ij_P_i_m_j_* or *βP*^2^*m*^2^.^28^

While no high temperature magnetization study has yet been performed to investigate the ferromagnetic Curie temperature (if indeed such a fixed temperature exists, in the case of cluster magnetism), a prediction of the behavior at this temperature may be possible. Ferromagnetic hysteresis exists at 300 K (Figure 3) and due to the coalescence of the FC and ZFC curves at this temperature, we might expect the ferromagnetism to cease at the first phase transition above room temperature. Alternatively, this order may occur in a relaxor-like manner across a temperature range, due to local fluctuations in the magnetic clusters. If the Landau expansion for this phase contains a prominent coupling term, some evidence of the ferromagnetic transition would be expected in the other order parameters. No large anomalies are seen in ether dielectric or elastic parameters between the ≈240 K rhombohedral–orthorhombic and the 475 K orthorhombic–tetragonal transition; with this in mind, it is possible that the ferromagnetic curie temperature could be anywhere up to the 475 K transition. The connection between the order parameters may facilitate stabilization of the magnetic order, allowing the ferromagnetism to persist to such a high temperature.

## 4. Conclusions

In this paper, we have provided a detailed study of three phase transitions in the room-temperature multiferroic Pb(Fe_0.5_Ta_0.5_)_0.4_(Zr_0.53_Ti_0.47_)_0.6_O_3_. A magnetic ordering temperature near 35-50 K that is suggestive of Fe spin clustering, a rhombohedral-orthorhombic transition near 240 K, and an orthorhombic-tetragonal transition near 465 K. Use of magnetic susceptibility, dielectric and elastic techniques allows a probe into the coupling between the different available types of order in this material.

RUS data have revealed a pattern of elastic softening just above room temperature which is attributed to the T ↔ O transition at ≈472 K and is closely similar to the pattern seen also a the same transition in BaTiO_3_ and KNN. Variations in elastic properties below ≈50 K, and also possibly below 90 K, can be understood quite simply in terms of (biquadratic) strain coupling at the proposed antiferromagnetic transition. The anomalies near 90 K may signal another structural transition, or precursor effects related to the ≈50 K transition. A 75 K hysteresis interval, the lack of a minimum in the shear modulus and slight stiffening with falling temperature below ≈235 K, are rather different from the patterns seen at the O ↔ R transition in BaTiO_3_ and KNN, however. Relatively high acoustic loss in the stability fields of the orthorhombic and tetragonal phases could be due to the mobility of ferroelastic twin walls, but may potentially be related also to clustering of ferromagnetic spins if these are coupled with strain.

Magnetization and dielectric examinations show anomalies at very nearly coincident temperatures to those found from elastic behavior, and these results, as well as previous findings such as magnetic switching of ferroelectric domains, suggest strong coupling between the multiple ferroic orders present in PZTFT. In two subsequent papers we will analyze similar results for Pb(Fe_0.5_Ta_0.5_)_0.3_(Zr_0.53_Ti_0.47_)_0.7_O_3_ and examine magnetoelectric switching data and dielectric anomalies near T_N_ for both the Ta and Nb isomorphs.
